# TRIM30α Is a Negative-Feedback Regulator of the Intracellular DNA and DNA Virus-Triggered Response by Targeting STING

**DOI:** 10.1371/journal.ppat.1005012

**Published:** 2015-06-26

**Authors:** Yanming Wang, Qiaoshi Lian, Bo Yang, Shanshan Yan, Haiyan Zhou, Lan He, Guomei Lin, Zhexiong Lian, Zhengfan Jiang, Bing Sun

**Affiliations:** 1 Institute of Biochemistry and Cell Biology, Shanghai Institutes for Biological Sciences, Chinese Academy of Sciences, Shanghai, China; 2 School of Life Sciences, University of Science and Technology of China, Hefei, China; 3 State Key Laboratory of Protein and Plant Gene Research, Key Laboratory of Cell Proliferation and Differentiation of the Ministry of Education, School of Life Sciences, Peking University, Beijing, China; Peking University-Tsinghua University Joint Center for Life Sciences, Beijing, China; 4 Key Laboratory of Molecular Virology & Immunology, Institute Pasteur of Shanghai, Chinese Academy of Sciences, Shanghai, China; University of California Berkeley, UNITED STATES

## Abstract

Uncontrolled immune responses to intracellular DNA have been shown to induce autoimmune diseases. Homeostasis regulation of immune responses to cytosolic DNA is critical for limiting the risk of autoimmunity and survival of the host. Here, we report that the E3 ubiquitin ligase tripartite motif protein 30α (TRIM30α) was induced by herpes simplex virus type 1 (HSV-1) infection in dendritic cells (DCs). Knockdown or genetic ablation of TRIM30α augmented the type I IFNs and interleukin-6 response to intracellular DNA and DNA viruses. *Trim30α*-deficient mice were more resistant to infection by DNA viruses. Biochemical analyses showed that TRIM30α interacted with the stimulator of interferon genes (STING), which is a critical regulator of the DNA-sensing response. Overexpression of TRIM30α promoted the degradation of STING via K48-linked ubiquitination at Lys275 through a proteasome-dependent pathway. These findings indicate that E3 ligase TRIM30α is an important negative-feedback regulator of innate immune responses to DNA viruses by targeting STING.

## Author Summary

Negative-feedback regulation is a broad and pivotal biological event to maintain the homeostasis of the host. Viral DNA species derived from DNA viruses or retroviruses can activate STING signaling to produce pro-inflammatory cytokines and type I interferon, which further recruit immune cells or induce interferon stimulated genes (ISGs) to clear viral infection respectively. However, excessive STING-signaling activation has been shown to induce autoimmune disorders. Thus, it is important to finely turn off STING signaling. Here we demonstrate that TRIM30α is rapidly induced followed by STING activation. *Trim30α*-deficient mice show more resistance to infection by DNA viruses. Meanwhile, knockdown or genetic ablation of TRIM30α augments the type I IFNs and IL-6 responses to intracellular DNA and DNA viruses. Biochemical analyses show that TRIM30α interacts with STING and promotes the degradation of STING via K48-linked ubiquitination at Lys275. These findings demonstrate that induced TRIM30α is a negative-feedback regulator of STING pathway activation triggered by DNA and DNA viruses, which helps the host to avoid excessive response and maintain homeostasis.

## Introduction

The innate immune system is the first barrier of defense against pathogens, and a variety of germline-encoded pattern recognition receptors (PRRs), including Toll-like receptors (TLRs), C-type lectin receptors (CLRs), RIG-I-like receptors (RLRs) and NOD-like receptors (NLRs), have evolved in this system [1]. PRRs recognize the pathogen-associated molecular patterns (PAMPs) of microbes, which activate the innate immune response. The major PAMPs include nucleic acids derived from diverse bacterial, viral and eukaryotic pathogens. Thus far, many RNA receptors have been verified, among which TLR3 and TLR7/8, which are located in endosomes, sense double-stranded (ds)RNA and single-stranded (ss)RNA [2,3]. Retinoic-acid-inducible gene I (RIG-I) and melanoma differentiation-associated gene 5 (MDA5), which are located in the cytoplasm, detect cytosolic RNA [4,5].

In the past 5 years, the molecular basis of DNA sensing by the innate immune system has begun to be revealed, and a variety of DNA receptors have been proposed [6]. For example, TLR9 senses the CpG motifs-contained DNA [7]. In addition, AIM2 (absent in melanoma 2) binds to DNA and activates the inflammasome complex via the adaptor protein ASC (apoptosis-associated speck-like protein containing a CARD) [8–10]. Furthermore, RNA polymerase III detects cytosolic DNA and induces type I interferons through the RIG-I pathway [11,12]. Moreover, many other DNA receptors, including DAI, IFI16, DDX41 and cGAS, trigger type I interferon response via adaptor STING [13–16]. Although aspects of the molecular basis of STING-mediated DNA sensing by the innate immune system have been determined, the precise mechanism by which this pathway is fine-tuned remains unclear. Normal activation of the DNA signaling pathway contributes to appropriate host defense. However, uncontrolled stimulation by DNA results in excessive production of inflammatory cytokines and type I IFNs, which may cause severe autoimmune diseases, such as systemic lupus erythematosus (SLE) [17]. Several negative regulators of the STING-meditated DNA signaling pathway have been described. For example, the E3 ubiquitin ligase TRIM21 induces the Lys48 (K48)-linked ubiquitination and degradation of DDX41 and negatively regulates the innate immune response to intracellular dsDNA [18]. Another E3 ubiquitin ligase, RNF5, which is constitutive expressed, inhibits virus-triggered signaling by targeting STING for ubiquitination and degradation [19]. Hiroyasu Konno et al demonstrated that cyclic dinucleotides (CDN) trigger the phosphorylation of STING by UNC-51-like kinase (ULK1), which inhibits STING activity and thus prevents the persistent transcription of innate immune genes [20]. A recent study has identified NLRC3 as another negative regulator of STING-induced innate immune response [21].

Previous work in our laboratory has demonstrated that TRIM30α negatively regulates TLR4 signaling by targeting TAB2 and TAB3 for degradation [22]. TRIM30α is also found to negatively regulate NLRP3 inflammasome activation by modulating reactive oxygen species production [23]. In this study, we report that TRIM30α is a negative-feedback regulator of innate immune responses to intracellular DNA and DNA viruses by promoting degradation of STING via K48-linked ubiquitination at Lys275.

## Results

### TRIM30α knockdown increases the host response to cytoplasmic DNA and DNA virus infection in D2SC cells

**Fig 1:**
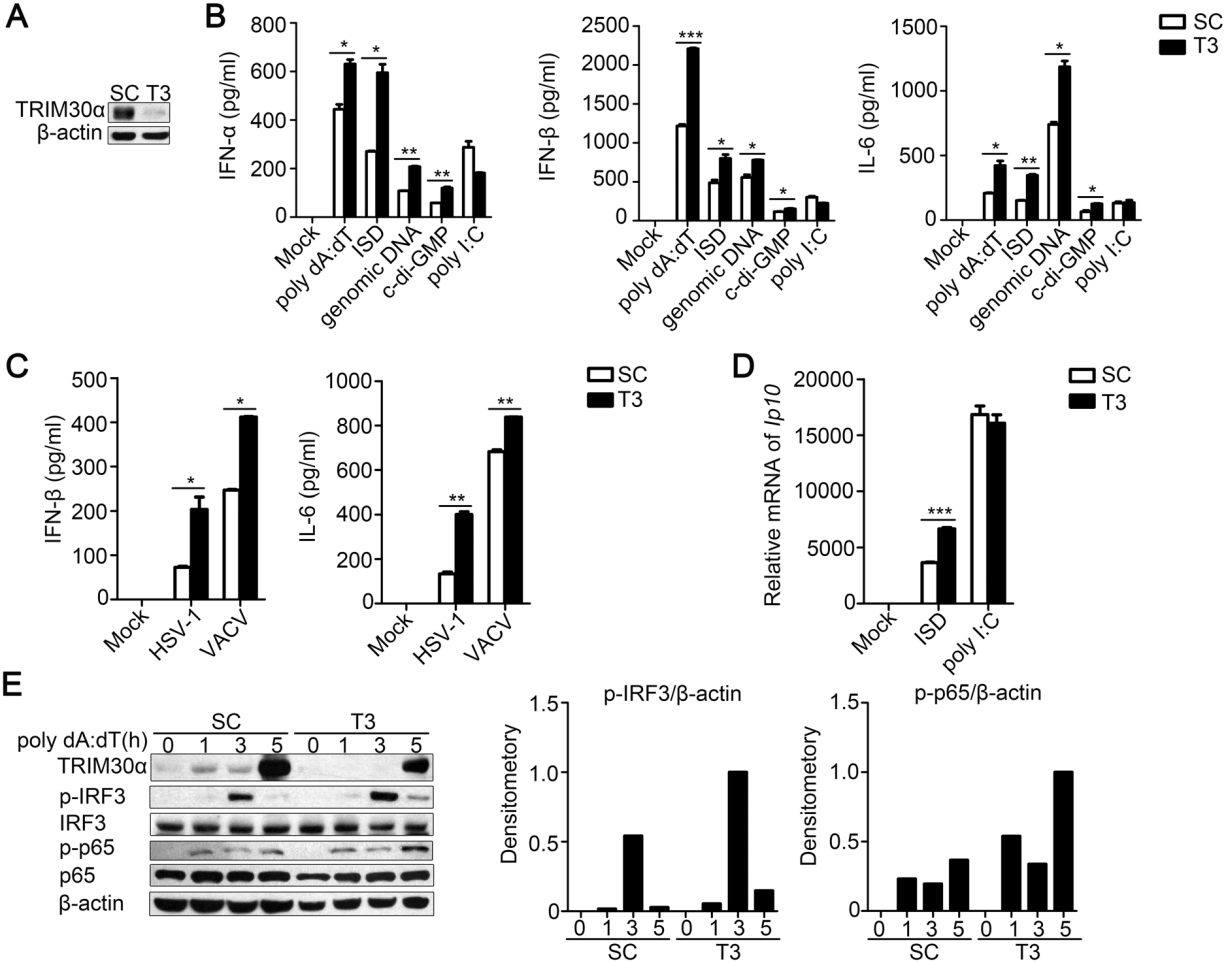
TRIM30α knockdown promotes immune signaling by cytoplasmic DNA and DNA virus infection in D2SC cells. (A) Immunoblot analysis of the knockdown of exogenous TRIM30α in D2SC cells treated with control siRNA (SC) or TRIM30α siRNA (T3) for 24 h and then stimulated for 16 h with poly(dA:dT) (1 μg/ml). β-actin served as a loading control throughout the experiment. (B, C) ELISA of type I IFN and IL-6 in D2SC cells treated with siRNA SC or T3 and then stimulated for 16 h with poly(dA:dT) (1 μg/ml), ISD (1 μg/ml), genomic DNA (2 μg/ml), c-di-GMP (8 μg/ml) or poly(I:C) (5 μg/ml) (C) or with HSV-1 (MOI 10) or vaccinia virus (VACV) (MOI 10). (D) Real-time PCR of IP-10 mRNA in D2SC cells treated with siRNA SC or T3 and then stimulated for 8 h with ISD (1 μg/ml) or poly(I:C) (5 μg/ml). (E) Immunoblot analysis of p-IRF3, p-p65 and TRIM30α in lysates of D2SC cells treated with siRNA SC or T3 and then stimulated for 1–5 h with poly(dA:dT) (1 μg/ml). Densitometry analysis to quantify ratio of p-IRF3 or p-p65 to β-actin is shown on the right. The data are representative of three independent experiments and are presented as mean ± SEM. *p < 0.05, **p < 0.01 and ***p < 0.001.

TRIM30α has been reported to be induced by TLR ligands in an NF-κB-dependent way and then negatively regulates TLR signaling by a ‘feedback’ mechanism. We found TRIM30α can also be induced upon intracellular poly(dA:dT) stimulation in bone marrow-derived dendritic cells (BMDCs) (S1A Fig). To address whether TRIM30α regulates the immune response to DNA-mediated signaling, we used TRIM30α siRNA (T3) to silence endogenous TRIM30α expression in D2SC cells, a mouse mDC cell line. The results showed that the T3 siRNA efficiently reduced the TRIM30α protein expression compared with control siRNA (SC) (Fig 1A). Various DNA ligands were used to stimulate D2SC cells, including poly(dA:dT), interferon stimulatory DNA (ISD), the genomic DNA of C57BL/6 mice and cyclic di-GMP (c-di-GMP). Knockdown of TRIM30α enhanced the production of type I IFN and IL-6 compared with controls (Fig 1B). In contrast, the induction of type I IFN by the synthetic analog of viral dsRNA poly (I:C) (the signaling of which is largely governed by MDA5) was reduced when TRIM30α was knocked down. To further confirm the function of TRIM30α in the anti-DNA viral response, we infected D2SC cells with the DNA viruses HSV-1 or vaccinia virus (VACV). The data showed that IFN-β and IL-6 production was dramatically increased in D2SC cells transfected with TRIM30α-specific siRNA (Fig 1C). TRIM30α knockdown also promoted the type I IFN and IL-6 expression at the mRNA level after stimulation with poly(dA:dT), ISD or HSV-1 (S1B and S1C Fig). We next examined the IFN-stimulated genes, such as the chemokine IP-10. Notably, TRIM30α knockdown enhanced the production of IP-10 mRNA upon ISD stimulation, but had no effect on the poly(I:C)-mediated signaling (Fig 1D).

Intracellular nucleic acids and most viruses activate NF-κB, which is indispensable for TRIM30α expression. We found that poly(I:C), poly(dA:dT), ISD, c-di-GMP, genomic DNA and DNA viruses, including HSV-1 and VACV, facilitated the expression of TRIM30α (S1D Fig). Moreover, TRIM30α expression was considerably decreased by treatment with two NF-κB inhibitors, the IκB kinase-2 (IKK-2) inhibitor TPCA-1 (2-[(aminocarbonyl) amino]-5-(4-fluorophenyl)-3-thio-phenecarboxamide) and PDTC (pyrrolidine dithiocarbamate), in BMDCs stimulated for 6 h with poly(I:C), ISD or HSV-1 (S1E Fig).

Type I IFN and IL-6 expression is dependent upon the transcription factors IRF3 and NF-κB. Therefore, the amount of phosphorylated-IRF3 and-p65 was determined in D2SC cells after exposure to poly(dA:dT) at different time points. We found that TRIM30α knockdown resulted in high level of phosphorylation of IRF3 and p65 after poly(dA:dT) treatment (Fig 1E). Collectively, these results indicate that specifically knocking down TRIM30α considerably enhances type I IFN and IL-6 activation and the expression of IP-10 in response to cytoplasmic DNA and DNA viruses.

### TRIM30α deficiency increases the host response to cytoplasmic DNA and DNA virus infection in dendritic cells

**Fig 2:**
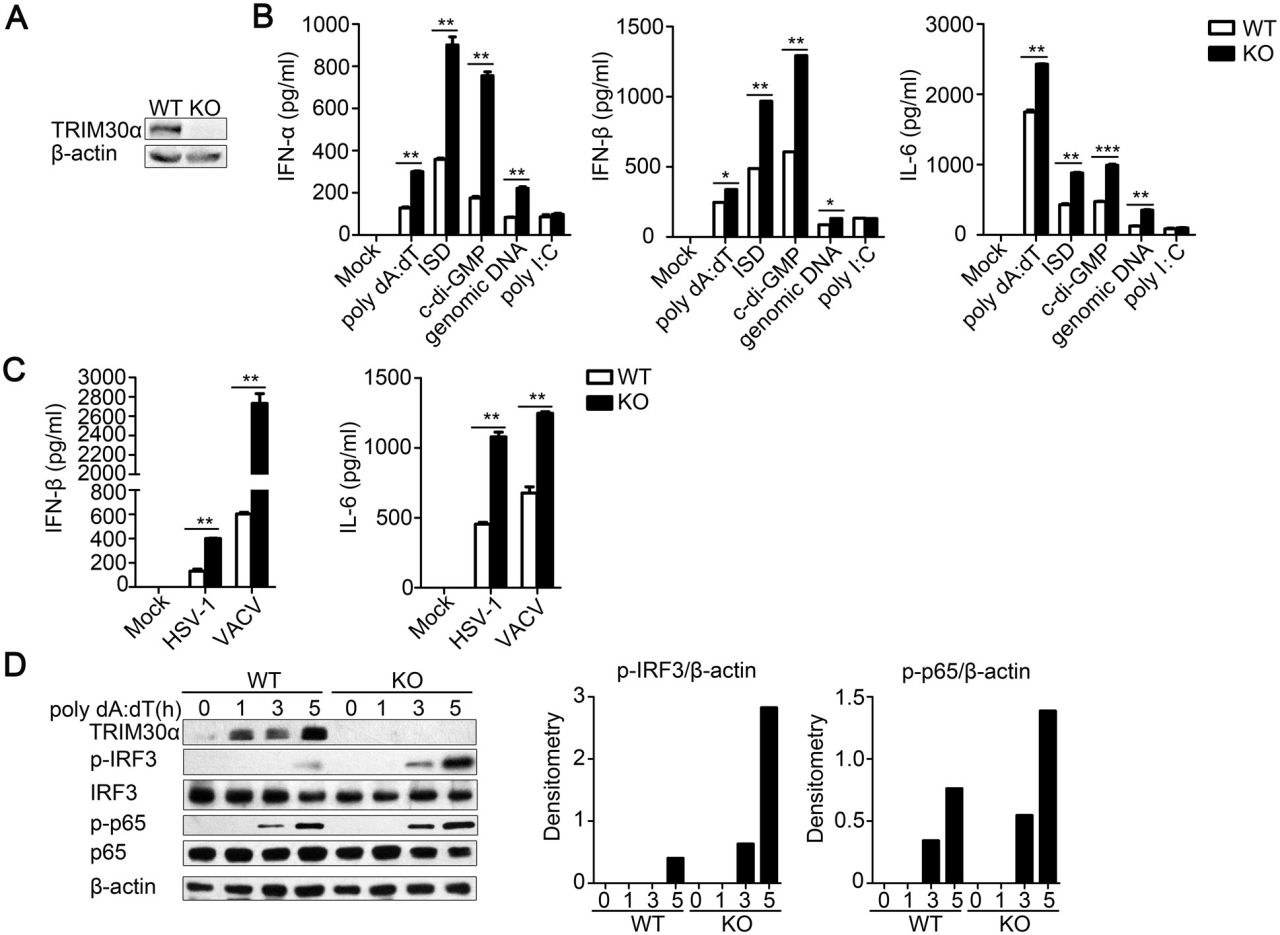
TRIM30α deficiency promotes immune signaling by cytoplasmic DNA and DNA virus infection in BMDCs. (A) Immunoblot analysis of TRIM30α expression in the splenocytes of wild type (WT) and *Trim30α* ^*-/-*^ (KO) mice. (B, C) ELISA of type I IFN and IL-6 in wild-type and *Trim30α* ^-/-^ BMDCs mock treated or stimulated for 16 h by transfection with poly(dA:dT) (1 μg/ml), ISD (1 μg/ml), c-di-GMP (8 μg/ml), genomic DNA (2 μg/ml) or poly(I:C) (5 μg/ml) (B); or with HSV-1 (MOI 10) or VACV (MOI 10) (C). (D) Immunoblot analysis of phosphorylated IRF3 (p-IRF3), p-p65 and TRIM30α in lysates of wild type and *Trim30α* ^-/-^ BMDCs stimulated for 1–5 h with poly(dA:dT) (1 μg/ml). Densitometry analysis to quantify ratio of p-IRF3 or p-p65 to β-actin is shown on the right. The data are representative of three independent experiments and are presented as mean ± SEM. *p < 0.05, **p < 0.01 and ***p < 0.001.

To further demonstrate the function of TRIM30α in immune response to DNA-mediated signaling, we generated *Trim30α*-deficient mice, in which the second exon was knocked out by homologous recombination (S2 Fig). Immunoblot analysis confirmed that TRIM30α expression was completely absent in *Trim30α*-deficient mice (Fig 2A). As shown in Fig 2B, TRIM30α deficiency resulted in much higher expression of type I IFN and IL-6 in response to multiple DNA ligands compared with wild-type DCs, similar to the results observed in D2SC cells. In contrast, TRIM30α deficiency did not affect poly(I:C) signaling (Fig 2B). Consistently with previous results, TRIM30α deficiency dramatically increased IFN-β and IL-6 expression induced by HSV-1 and VACV infection at protein level (Fig 2C). Beside the role in DNA sensing pathway, we also determine the function of TRIM30α against RNA viruses. As shown in S3 Fig, TRIM30α knockdown or deficiency dramatically potentiated IFN-β and IL-6 production by infection with VSV in D2SC cells and BMDCs (S3 Fig). The results suggest that TRIM30α negatively regulate immune response both to DNA and RNA viruses.

Finally, we assessed IRF3 and p65 phosphorylation in BMDCs after poly(dA:dT) stimulation and found that TRIM30α deficiency promoted much higher expression of the phosphorylation of endogenous IRF3 and p65 (Fig 2D). These results suggest that TRIM30α plays a negative role in regulating type I IFN and IL-6 production in response to cytoplasmic DNA and DNA viruses.

### TRIM30α deficiency inhibits HSV-1 infection

**Fig 3:**
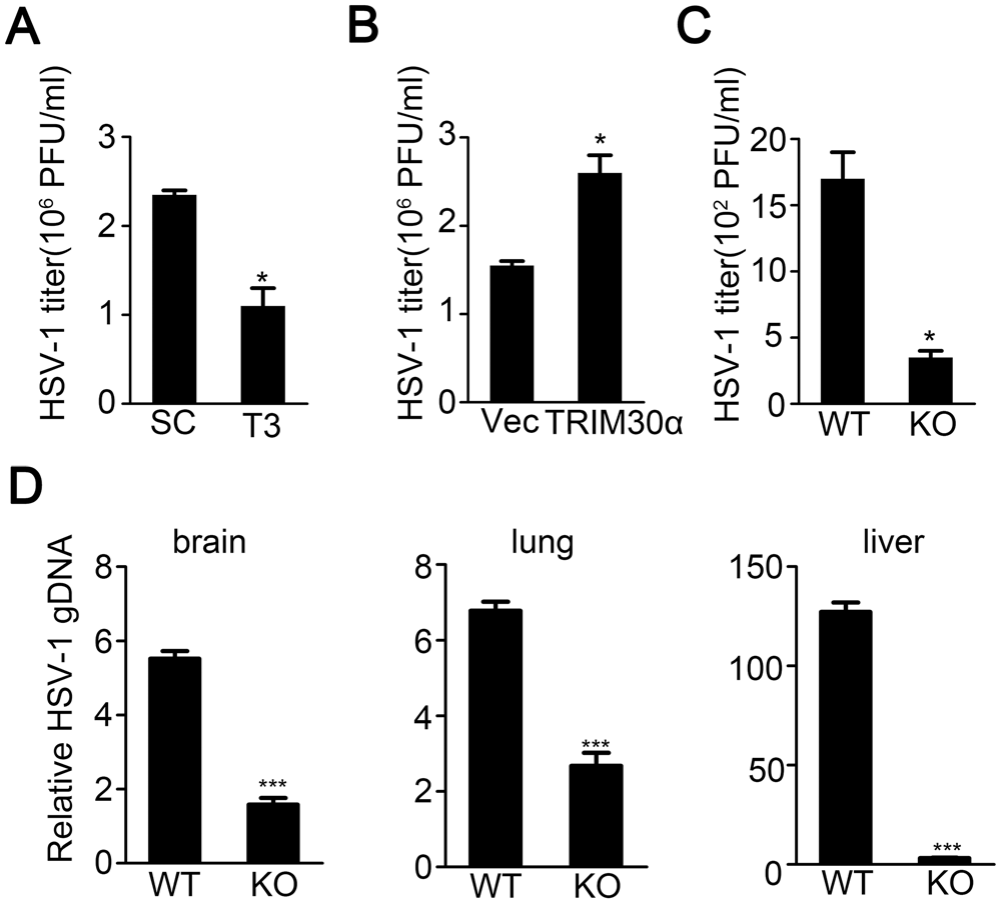
TRIM30α deficiency inhibits DNA virus infection. (A, B) Viral titers in L929 cells transfected with control siRNA SC and T3 (A), empty vector (Vec) or TRIM30α plasmid (B) for 24 h and then infected with HSV-1 (MOI 10) for 20 h. The titers of HSV-1 were determined by standard plaque assay. (C) Viral titers in wild type and *Trim30α* ^-/-^ mice intraperitoneally injected with HSV-1 (1×10^7^ plaque-forming units (PFU)). HSV-1 titers were measured 20 h later by plaque assay of peritoneal wash fluid. (D) Real-time PCR of HSV-1 genomic DNA in the brain, lung and liver from wild type and *Trim30α* ^-/-^ mice infected with HSV-1 (2×10^7^ PFU) (i.v.) for 2 days. The data are representative of three independent experiments and are presented as mean ± SEM. *p < 0.05, ***p < 0.001.

To evaluate the function of TRIM30α in host antiviral responses in vitro, we knocked down the expression of TRIM30α in mouse fibroblast L929 cells and then infected these cells with the DNA virus HSV-1. As expected, TRIM30α knockdown suppressed HSV-1 infection (Fig 3A). In contrast, overexpression of TRIM30α markedly facilitated HSV-1 replication (Fig 3B). These data suggest that TRIM30α is an important negative regulator in anti-viral defense. To further identify the role of TRIM30α in vivo host defense, wild type and *Trim30α*-deficient mice were challenged by intraperitoneal (i.p.) injection with HSV-1, and virus titers were examined 20 h later. Significantly more HSV-1 replication was detected in wild type mice (Fig 3C).

Moreover, genomic DNA copies of HSV-1 were dramatically reduced in the brain, lung and liver of *Trim30α*-deficient mice in comparison to wild type mice upon HSV-1 infection for 2 days (Fig 3D). Interestingly, the liver showed much more difference between WT and KO mice compared to brain and lung, which was worthy to be explored. Together these data suggest that TRIM30α knockdown or deficiency inhibited HSV-1 replication both in vitro and in vivo.

### TRIM30α deficiency protects mice from infection with HSV-1

**Fig 4:**
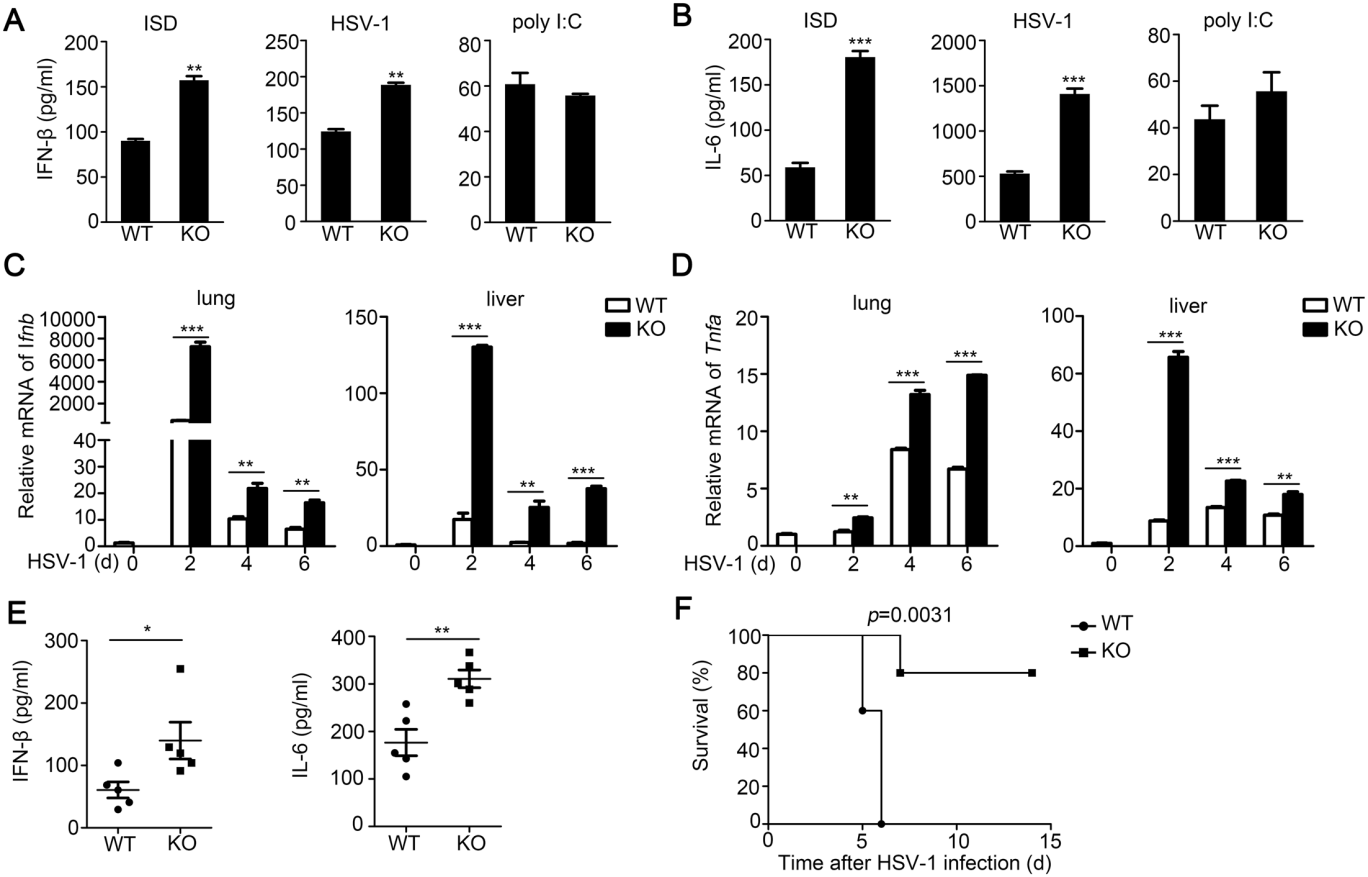
TRIM30α deficiency protects the mice from DNA virus infection. (A, B) ELISA of IFN-β and IL-6 in wild-type and *Trim30α* ^-/-^ CD11c^+^ splenocytes stimulated for 24 h with ISD (1 μg/ml), HSV-1 (MOI 10) or poly(I:C) (5 μg/ml). (C, D) Real-time PCR of IFN-β and TNF-α in the lung and liver from wild type and *Trim30α* ^-/-^ mice infected with HSV-1 (2×10^7^ PFU) (i.v.) for the indicated times. (E) ELISA of IFN-β and IL-6 in serum of wild type and *Trim30α* ^-/-^ mice 6 h after intravenous infection with HSV-1 (1.2×10^7^ PFU) (n = 5). (F) Survival of age- and sex-matched wild-type and *Trim30α* ^*-/-*^ mice (n = 5 per group) infected with HSV-1 (2×10^7^ PFU) (i.v.) and monitored daily for 15 d. The data are representative of three independent experiments and are presented as mean ± SEM. *p < 0.05, **p < 0.01 and ***p < 0.001.

To investigate the role of TRIM30α in host antiviral innate response, we isolated CD11c^+^ splenocytes from wild-type and *Trim30α*-deficient mice and treated these cells for 16 h with ISD, HSV-1 or poly(I:C). The *Trim30α*-deficient CD11c^+^ splenocytes produced high levels of IFN-β and IL-6 upon stimulation with ISD and HSV-1. However, TRIM30α deficiency had little effect on poly(I:C)-triggered response (Fig 4A and 4B). Besides DCs, macrophages are also involved in immune response triggered by intracellular DNA or DNA virus. We then obtained peritoneal macrophages (PM) from wild type and *Trim30α* ^-/-^ mice and then the above PM were stimulated with ISD or infected with HSV-1. The real-time PCR analysis demonstrated that *Trim30α* ^-/-^ PM produced more type I IFN and ISGs, suggesting that the negative role of TRIM30α in regulating DNA-sensing signaling pathway was not restricted in DCs (S4A and S4B Fig).

To further elucidate the function of TRIM30α in vivo, wild-type and *Trim30α*-deficient mice were infected intravenously (i.v.) with HSV-1. Then we isolated various organs to evaluate immune response induced by DNA viruses. The levels of IFN-β and TNF-α mRNAs in liver and lung were markedly potentiated in *Trim30α*-deficient mice (Fig 4C and 4D). In addition, we observed higher IFN-β and IL-6 levels in the serum of *Trim30α*-deficient mice than in wild-type mice followed by HSV-1 infection (Fig 4E). Survival experiments demonstrated that the survival rate was significantly prolonged in the *Trim30α*-deficient mice infected with HSV-1 (Fig 4F). Thus, TRIM30α negatively controls DNA virus-triggered signaling, and TRIM30α deficiency protects mice from DNA virus infection.

### TRIM30α interacts with STING

**Fig 5:**
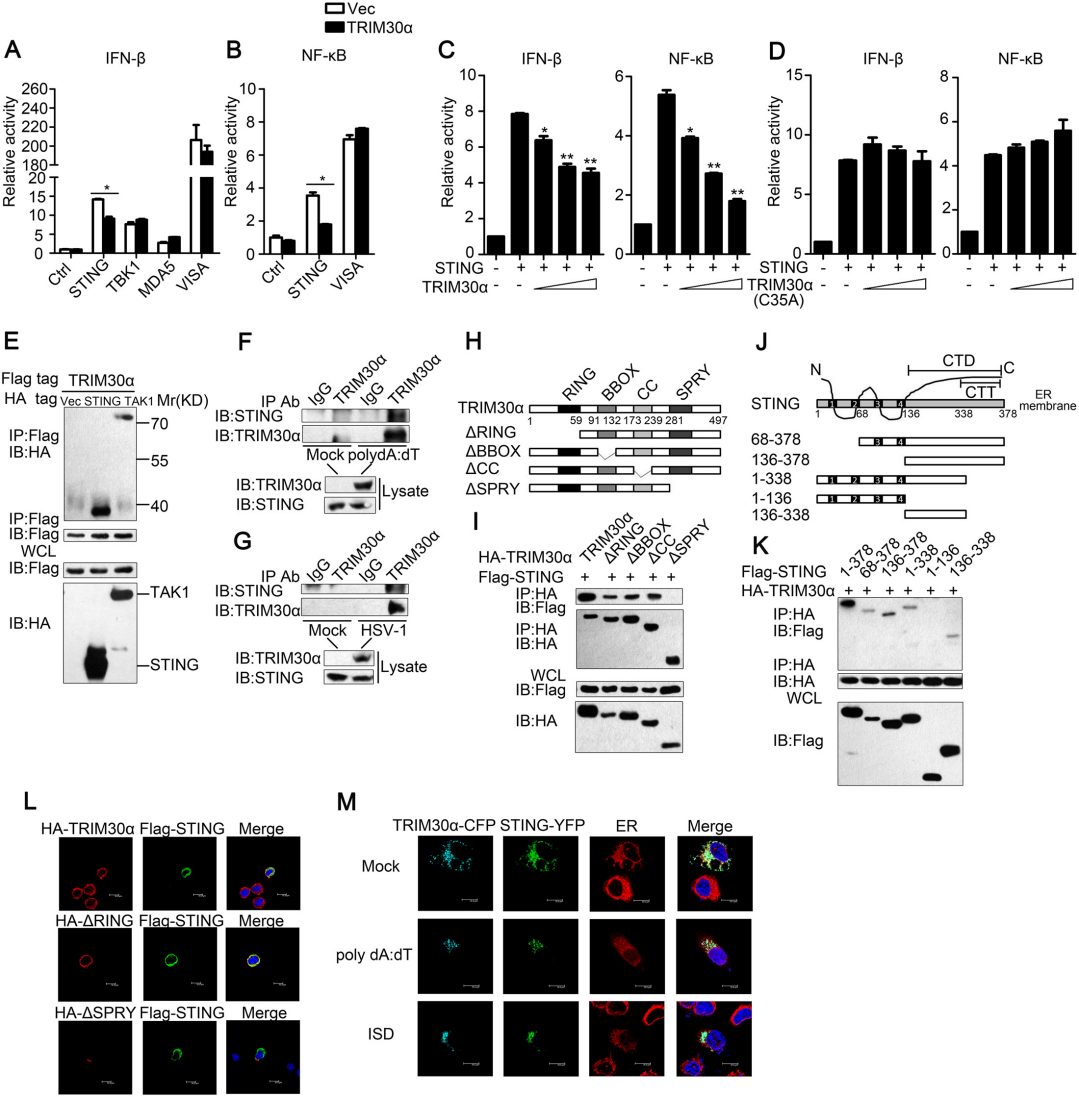
TRIM30α interacts with STING. (A) Luciferase activity of MEF cells transfected with the IFN-β luciferase reporter, together with STING, TBK1, MDA5 or VISA, and the empty vector (Vec) or the TRIM30α plasmid. (B) Luciferase activity of MEF cells transfected with the NF-κB luciferase reporter, together with STING or VISA, and the empty vector (Vec) or the TRIM30α plasmid. (C and D) Luciferase activity of MEF cells cotransfected with the IFN-β promoter or the NF-κB promoter and empty vector or increasing amounts of TRIM30α (C) or C35A (D) (0.2, 0.4 and 0.6 μg) together with STING. (E) Immunoblot analysis of lysates from HEK293T cells transfected with Flag-TRIM30α together with HA-tagged vector, STING or TAK1 plasmids, followed by immunoprecipitation (IP) with anti-Flag Ab and immunoblot analysis with anti-HA Ab. (F, G) Immunoblot analysis in lysates of D2SC cells mock treated or stimulated with poly(dA:dT) (1 μg/ml) (F); or with HSV-1 (MOI 10) (G) for 8 h, followed by immunoprecipitation with anti-TRIM30α Ab and immunoblot analysis with anti-STING Ab. (H) A schematic presentation of full-length TRIM30α and its mutants. RING, ring-finger domain; BBOX, B-box domain; CC, coiled-coil domain; SPRY, SPRY domain. (I) Immunoblot analysis of lysates from HEK293T cells transfected with the indicated plasmids, followed by immunoprecipitation (IP) with anti-HA Ab and immunoblot analysis with anti-Flag Ab. (J) A schematic presentation of full-length STING and its mutants. (Transmembrane) TM1, 21-41aa; TM2, 47-67aa; TM3, 87-106aa; TM4, 115-135aa; CTD, carboxy-terminal domain; CTT, carboxy-terminal tail. (K) Immunoblot analysis of lysates from HEK293T cells transfected with the indicated plasmids and then performed as in I. (L) Confocal microscopy of L929 cells transfected for 24 h with Flag-tagged STING and HA-tagged full-length TRIM30α, ΔRING or ΔSPRY mutant of TRIM30α. Immunofluorescence was performed using anti-HA (red) and anti-Flag (green). (M) Confocal microscopy of L929 cells transfected for 24 h with cyan fluorescent protein-labeled TRIM30a (CFP-TRIM30α) and yellow fluorescent protein-labeled STING (YFP-STING) and then mock treated or stimulated for 4 h with poly(dA:dT) (1 μg/ml) or ISD (1 μg/ml). Nuclei were stained with the DNA-intercalating dye DAPI. Staining of calnexin served as a marker of the endoplasmic reticulum (ER). The data are representative of three independent experiments and are presented as mean ± SEM. *p < 0.05 and **p < 0.01.

To identify the molecular mechanisms by which TRIM30α inhibits the DNA-triggered response, luciferase assays were performed to detect the target of TRIM30α. We found that TRIM30α inhibited the IFN-β reporter activation mediated by STING, a key adaptor protein for most DNA-sensing pathways [24,25]. However, TRIM30α did not affect the downstream kinase of STING, TBK1, and had no influence on MDA5- and virus-induced signaling adaptor (VISA)-induced signaling (Fig 5A). In addition, TRIM30α overexpression significantly inhibited STING-induced NF-κB reporter activation but did not affect VISA (Fig 5B). It is hypothesized that TRIM30α targets STING to inhibit DNA-mediated response. As shown in Fig 5C, exogenous expression of TRIM30α inhibited STING-induced IFN-β and NF-κB reporter activation in a dose dependent manner (Fig 5C). In contrast, TRIM30α (C35A), which contains an enzymatically inactive mutant of the RING domain had no effect on STING-induced IFN-β and NF-κB reporter activation (Fig 5D). All these suggest that TRIM30α suppresses STING signaling dependent on its RING domain. Furthermore, co-immunoprecipitation assays were performed and the results showed that TRIM30α interacted with STING when co-transfected into HEK293T cells (Fig 5E). TGF beta-activated kinase (TAK1) interacts with TRIM30α, which has been previously reported, was used as a positive control [22]. We next performed endogenous co-immunoprecipitation experiments and verified that endogenous TRIM30α could interact with STING in D2SC cells after poly(dA:dT) or HSV-1 stimulation (Fig 5F and 5G). To further demonstrate the interaction, TRIM30α and STING were quickly translated in vitro, and immunoprecipitation analysis indicated that TRIM30α could interact with STING directly (S5 Fig).

To explore the domains that govern the association between TRIM30α and STING, HA-tagged full-length TRIM30α and various TRIM30α truncations were expressed in HEK293T cells. Co-immunoprecipitation assays showed that only the SPRY domain of TRIM30α bound to STING (Fig 5H and 5I). Previous studies have reported that STING is a multiple transmembrane domain-containing protein and that the C-terminal amino acids of STING include a globular carboxy-terminal domain (CTD, amino acids 136–378) and carboxy-terminal tail (CTT, amino acids 338–378) [26,27]. We found that only the C-terminal amino acids 136–338 of STING were required for interaction with TRIM30α (Fig 5J and 5K). Flag-tagged STING, HA-tagged TRIM30α and TRIM30α truncations were expressed in L929 cells. Confocal microscopy experiments showed that STING was co-localized with full-length TRIM30α and ΔR, but not ΔSPRY, confirming that the SPRY domain of TRIM30α is required for interaction with STING (Fig 5L). We then expressed CFP-TRIM30α and YFP-STING in L929 cells and detected the co-localization of these two proteins (Fig 5M). Interestingly, it was also found that STING translocated from the endoplasmic reticulum (ER) to perinuclear sites in response to poly(dA:dT) and ISD, in agreement with previous studies [25]. These results suggest that TRIM30α interacts with STING to inhibit DNA-mediated immune response.

### TRIM30α enhances the degradation of STING

**Fig 6:**
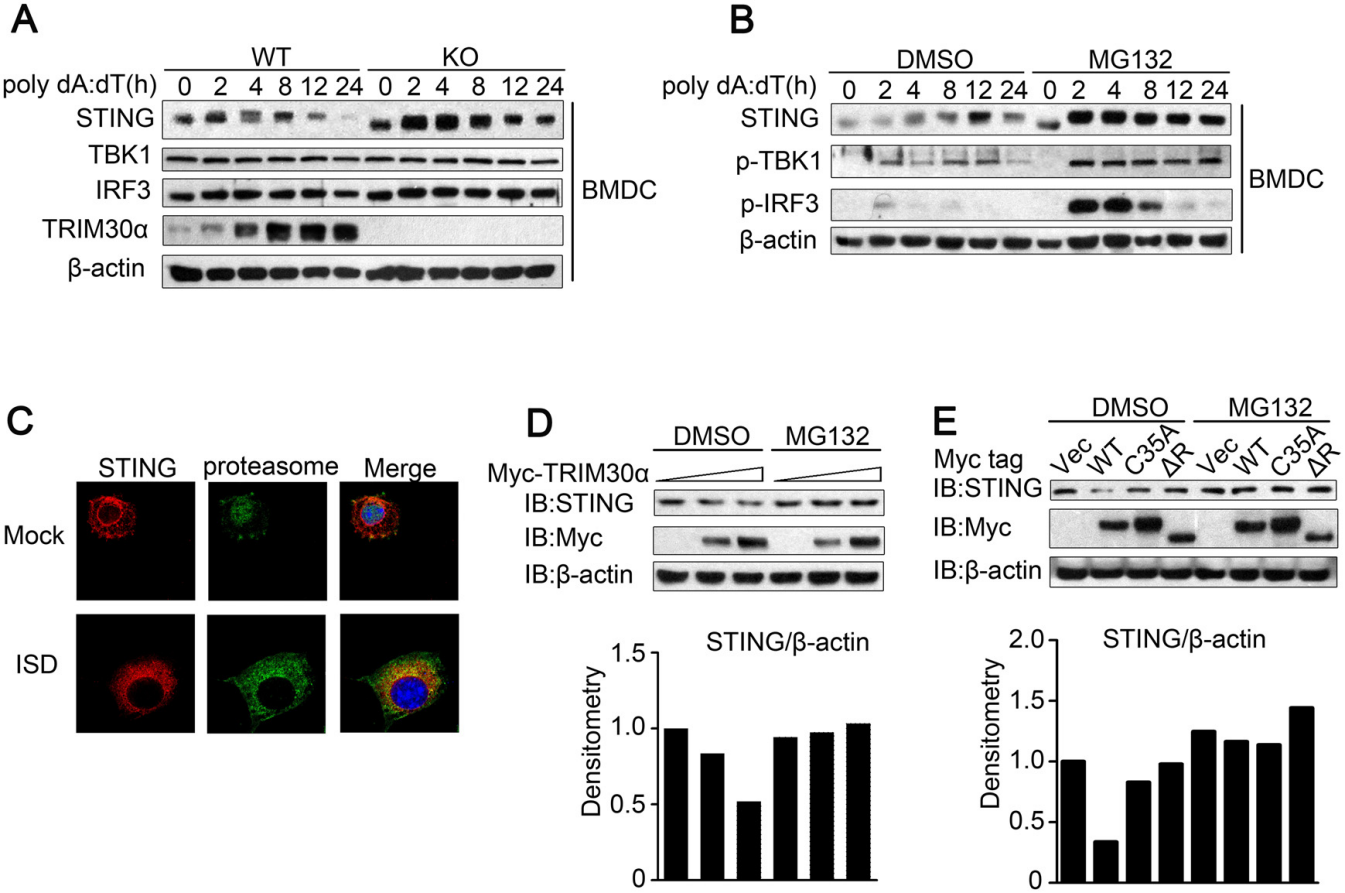
TRIM30α enhances the degradation of STING. (A) Immunoblot analysis of STING, TBK1, IRF3 and TRIM30α in lysates from wild type and *Trim30α* ^-/-^ BMDCs stimulated for 2–24 h with poly(dA:dT) (1 μg/ml). (B) Immunoblot analysis of STING in lysates from wild type BMDCs stimulated for 2–24 h with poly(dA:dT) (1 μg/ml) in the presence or absence of 20 mM MG132. (C) Confocal microscopy of L929 cells transfected for 24 h with Flag-tagged STING and then mock treated or stimulated for 12 h with ISD (1 μg/ml). Immunofluorescence was performed using anti-Flag (red) and anti-20S proteasome β1 (green). Nuclei were stained with the DNA-intercalating dye DAPI. (D) Immunoblot analysis of STING in lysates of L929 cells transfected with increasing doses of Myc-tagged TRIM30α (0, 0.8 and 1.2 μg) and then treated for 6 h with DMSO (negative control) or 20 mM MG132. Densitometry analysis to quantify ratio of STING to β-actin is shown on the below. (E) Immunoblot analysis of STING in lysates of L929 cells transfected with full-length TRIM30α, C35A or ΔR (1μg) and then treated as in D.

It is very important to determine the mechanism by which interaction of TRIM30α with STING suppress the intracellular DNA-sensing pathway. As shown in Fig 6A, *Trim30α*-deficient BMDCs maintained a higher expression of STING than wild type BMDCs after poly(dA:dT) stimulations of different durations (Fig 6A). In contrast, the expression of TBK1 and IRF3, two adaptors downstream of STING, maintained unchanged in *Trim30α*-deficient BMDCs, suggesting that TRIM30α may suppress the stability of STING. We next treated wild type BMDCs with poly(dA:dT) in the presence or absence of MG132, the inhibitor of proteasome. We observed a high level expression of STING in BMDCs upon MG132 treatment than control, indicating that STING undergoes degradation after stimulation with intracellular DNA in a proteasome way (Fig 6B). Moreover, MG132 treatment significantly promoted much higher expression of phosphorylation of TBK1 and IRF3 than control cells, which mimics TRIM30α deficiency upon intracellular DNA stimulation. Confocal microscopy further demonstrated that STING was co-localized with proteasome during ISD stimulation, suggesting that STING may undergo degradation via proteasome in response to DNA stimulus (Fig 6C).

Because TRIM30α promotes the degradation of TAB2 and TAB3 [22], we hypothesized that TRIM30α also promotes the degradation of STING. To address this, TRIM30α was overexpressed in L929 cells. The expression of endogenous STING in L929 cells was diminished in a dose-dependent manner and rescued by MG132 (Fig 6D). Furthermore, we determined whether STING degradation is dependent upon the RING domain of TRIM30α, which confers it E3 ubiquitin ligase activity. TRIM30α (C35A) and TRIM30α (ΔR) attenuated STING degradation when expressed in L929 cells compared with full-length TRIM30α (Fig 6E). Collectively, these results indicated that the interaction of TRIM30α with STING enhances STING degradation in a proteasome pathway, which is dependent on its RING domain.

### TRIM30α targets STING for K48-linked ubiquitination at Lys275

**Fig 7:**
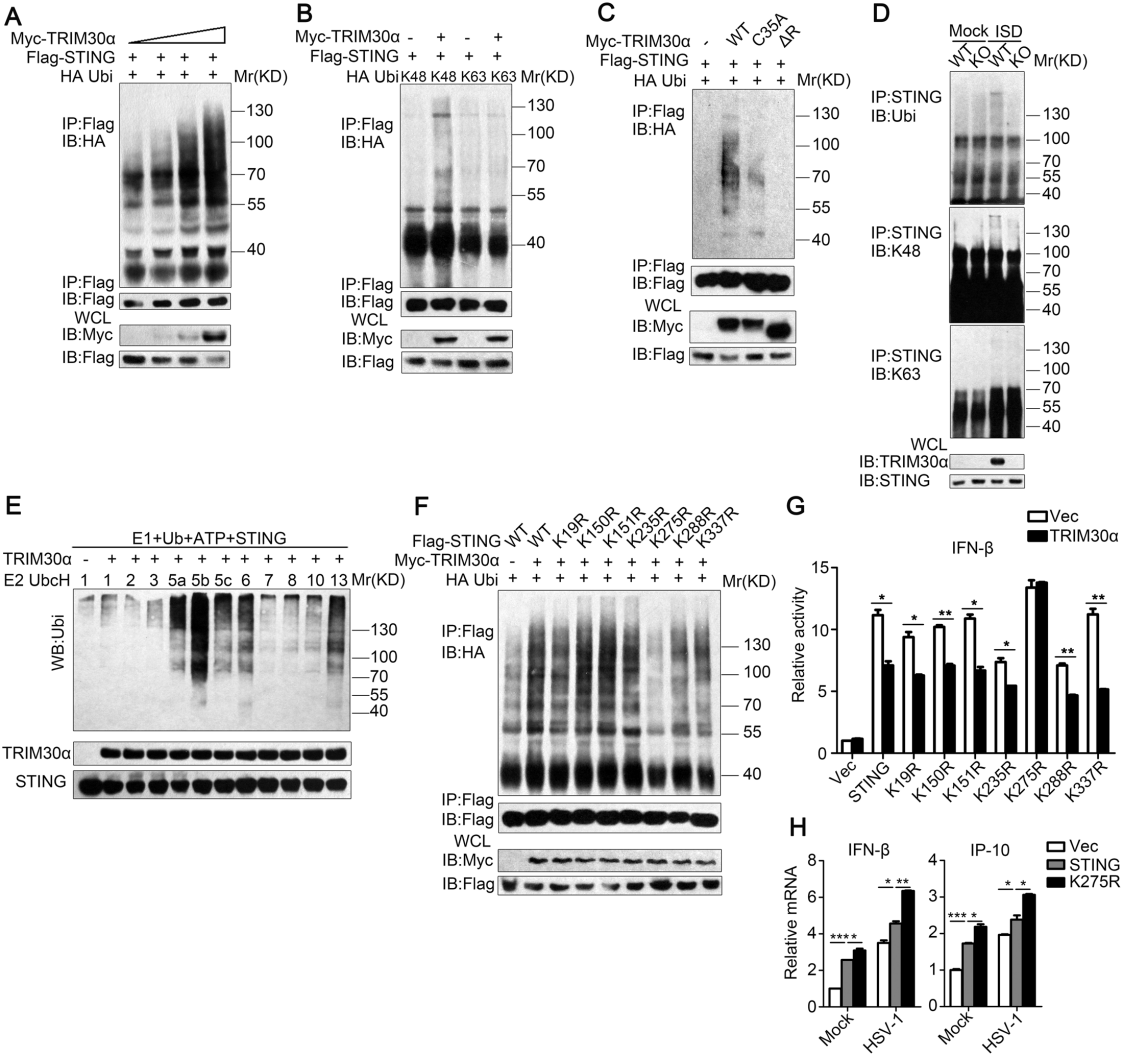
TRIM30α is an E3 ubiquitin ligase and targets STING for K48-mediated ubiquitination at Lys275. (A) Immunoblot analysis of lysates from HEK293T cells transfected with plasmids for Flag-STING, HA-ubiquitin and increasing concentrations of Myc-TRIM30α (0, 1, 1.5 and 2 μg), followed by immunoprecipitation with anti-Flag, and analyzed via immunoblot with anti-HA Ab. (B) Immunoblot analysis of lysates from HEK293T cells transfected with various combinations of plasmids for Myc-TRIM30α, Flag-STING, HA-K48-ubi and HA-K63-ubi and then performed as in A. (C) Immunoblot analysis of lysates from HEK293T cells transfected with the indicated plasmids and Myc-tagged TRIM30α (C35A), ΔR and then performed as in B. (D) Immunoblot analysis of lysates in wild-type and *Trim30α* ^-/-^ BMDCs stimulated for 8 h with ISD (1 μg/ml), followed by immunoprecipitation with anti-STING Ab and analyzed with anti-Ub, anti-K48- and anti-K63-linked ubiquitin. (E) Immunoblot analysis of STING ubiquitination in vitro. TRIM30α and STING were quickly translated in vitro, and then, the biotin-ubiquitin E1 and indicated E2s were added for the ubiquitination assays. Ubiquitination was assessed by anti-ubi. (F) Immunoblot analysis of lysates from HEK293T cells transfected with Myc-tagged TRIM30α, Flag-tagged wild-type and mutant STING together with HA-tagged ubiquitin, then performed as in A. (G) Luciferase assays of MEF cells cotransfected with the IFN-β promoter and empty vector or TRIM30α together with wild-type and mutant STING plasmids. (H) Real-time PCR of IFN-β and IP-10 mRNA in L929 cells transfected with Flag-tagged vector, wild type STING and K275R mutant, 24 h after transfection, then infected the above cells with HSV-1 (MOI 10) for 10 h. The data are representative of three independent experiments and are presented as mean ± SEM. *p < 0.05, **p < 0.01 and ***p < 0.001.

Most TRIM proteins contain RING domains, which allow them to mediate ubiquitylation events [28]. Therefore, we hypothesized that TRIM30α is an E3 ubiquitin ligase for STING. To address this, TRIM30α and STING were co-transfected in HEK293T cells. Immunoprecipitation and immunoblot analysis showed that TRIM30α could ubiquitinate STING in a dose-dependent manner (Fig 7A). Moreover, we observed that TRIM30α overexpression enhanced K48-linked ubiquitination of STING but not K63-linked ubiquitination (Fig 7B). K48-linked ubiquitination is normally linked to proteasomes-mediated degradation of proteins, which is in line with our early data (Fig 6D and 6E). As shown in Fig 7C, TRIM30α (C35A) and ΔR dramatically attenuated the ubiquitination of STING compared with full-length TRIM30α, suggesting that TRIM30α ubiquitinates STING dependent upon its RING domain activity (Fig 7C). Next, we examined the ubiquitination of STING in primary cells and observed that wild type and K48-linked ubiquitination of STING in wild type BMDCs was increased in comparison to *Trim30α*-deficient BMDCs stimulated with ISD for 8 h (Fig 7D). Collectively, these findings indicate that TRIM30α is an E3 ubiquitin ligase for STING and mediates STING degradation via the proteasome pathway. To further investigate whether TRIM30α directly ubiquitinates STING, TRIM30α and STING were quickly translated in vitro. In vitro ubiquitination showed that the TRIM30α protein directly mediated ubiquitination of STING (Fig 7E).

In order to map the ubiquitination sites on STING that are targeted by TRIM30α, we replaced each of the seven STING lysine (K) residues that not in transmembrane region with arginine (R). We then expressed TRIM30α, wild-type and mutant STING in HEK293T cells. As shown in Fig 7F, the ubiquitination of mutation of K275 to arginine was obviously attenuated (Fig 7F). In addition, luciferase assays showed that TRIM30α significantly suppressed wild-type and STING mutants-induced IFN-β reporter activity except for STING K275R (Fig 7G). Moreover, STING K275R maintained high level in HSV-1 triggered IFN-β and IP-10 production in comparison to wild type STING (Fig 7H). Taken together, the results suggest that Lys275 was the ubiquitination site of STING targeted by TRIM30α.

## Discussion

STING has been identified as an essential adaptor protein for controlling TLR-independent cytosolic DNA signaling [29]. Cytosolic DNA derived from DNA viruses, bacteria or parasites is sensed by DNA receptors that subsequently activate STING. Activated STING recruits and activates the cytosolic kinases IKK and TBK1, which in turn activate NF-κB and IRF3, respectively [30]. In addition, cyclic dinucleotides (CDNs) can directly bind and activate STING. However, the regulation of STING signaling remains to be fully elucidated. In this context, we discovered the novel function of TRIM30α in negatively regulating the STING pathway. TRIM30α deficiency or knockdown enhanced the production of type I IFNs and the inflammatory cytokine IL-6 upon stimulation with intracellular DNA or infection with the DNA viruses HSV-1 or VACV in BMDCs and D2SC cells. Moreover, *Trim30α-*deficient mice were resistant to HSV-1 compared with wild type mice. In vitro and in vivo studies demonstrated the negative role of TRIM30α in cytosolic DNA-mediated responses. Co-immunoprecipitation and immunoblot analyses indicated that TRIM30α interacted with STING through binding of the SPRY domain of TRIM30α with C-terminal amino acids 136–338 of STING. We further investigated that TRIM30α mediated K48-linked ubiquitination of STING at Lys275, which promoted STING degradation via the proteasome-dependent pathway. In our experiments, we found that TRIM30α expression was induced by DNA virus-triggered NF-κB activation, which then induced STING degradation, and that these events negatively regulated the STING-mediated signaling pathway.

Our laboratory has previously shown that TRIM30α expression is dependent on NF-κB activation and then targeting of TAB2 and TAB3 for degradation, thus attenuating the TLR signaling pathway [22]. Based on our previous work, we speculate that TRIM30α may act as a brake to prevent excessive immune response activation. In TLR and STING-mediated signaling, TRIM30α serves as an important feedback regulator and appropriately controls excessive inflammatory responses or type I IFNs production. Our results suggest that TRIM30α may be a negative regulator involved in other immune pathways in general. This hypothesis remains to be explored.

In the present study, we determined that TRIM30α is an E3 ubiquitin ligase for STING, the activity of which is dependent upon its RING domain. However, TRIM30α induces TAB2 degradation via lysosomes but not the ubiquitin-proteasome pathway. This observation suggests that TRIM30α is a multifunctional protein, degrading different substrates via distinct pathways. However, the characteristics of the target elements and the manner by which TRIM30α chooses the degradation pattern remain unclear.

Once activated, STING leads to increased expression of inflammatory cytokines and/or type I IFNs. Therefore, prevention of STING activity is of utmost importance for avoiding severe autoimmune disorders. Glen N. Barber demonstrated that when STING undergoes autophagy-dependent delivery, it is phosphorylated by serine/threonine UNC-51-like kinase [20]. Phosphorylation of Ser366 in STING was found to inhibit STING-dependent IRF3 activity but not NF-κB activity. However, TRIM30α can efficiently attenuate both the IRF3 and NF-κB pathways, dampening persistent immune activation. TRIM30α is a fine-tuned regulator in the down-regulation of STING-mediated signaling.

## Materials and Methods

### Ethics statement

C57BL/6 mice were purchased from the Shanghai Laboratory Animal Center (SLAC). All mice were bred and kept in specific pathogen-free (SPF) conditions in the Shanghai Institute of Biochemistry and Cell Biology. All animal care and use protocols were performed in accordance with the Regulations for the Administration of Affairs Concerning Experimental Animals approved by the State Council of People's Republic of China. The animal experiments were approved by the Institutional Animal Care and Use Committee of the Shanghai Institute of Biochemistry and Cell Biology, Chinese Academy of Sciences (Approval Number: IBCBSPF0028).

### Mice

*TRIM30α*-deficient mice were generated on a 129 background and backcrossed to C57BL/6 for at least 7 generations by the Shanghai Research Center for Model Organisms. Mice 6–8 weeks of age that were matched by body weight and sex were used in the experiments.

### Reagents and cDNA constructs

The poly(I:C), ISD and c-di-GMP were from Invitrogen. Poly(dA:dT) and lipopolysaccharide (LPS) were from Sigma. Genomic DNA from C57BL/6 mice was made in-house. The following antibodies were used for immunoblot analysis or immunoprecipitation: anti-HA (CO-MMS-101R; Covance), anti-Flag (F3165; Sigma), anti-STING (3337; Cell Signaling), anti-TRIM30α (previously described) [22], K63-specific anti-ubiquitin (05–1313; Millipore), K48-specific anti-ubiquitin (05–1307; Millipore), anti-IRF3 (sc-9082; Santa Cruz), anti-phosphorylated IRF3 (4947s; Cell Signaling), anti-p65 (4764s; Cell Signaling) and anti-phosphorylated p65 (3033s; Cell Signaling). The following antibodies were used for confocal microscopy: anti-20S proteasome β1 (SC-67345; Santa Cruz); anti-calnexin (C4731; Sigma) and Alexa Fluor 647 donkey anti-rabbit IgG (711-175-152; Molecular Probes). Lipofectamine 2000 was obtained from Invitrogen. The X-tremeGENE DNA transfection reagent was acquired from Roche. The Turbofect transfection reagent was acquired from ThermoFisher. The TNT Quick-coupled Transcription/Translation Systems kit was obtained from Promega. The ubiquitination kit (BML-uw9920-0001) was purchased from Enzo Life Sciences. The Protein A/G Plus-Agarose immunoprecipitation reagent was purchased from Santa Cruz. Anti-HA beads were purchased from Sigma. The ELISA kits were acquired from the following sources: murine IFN-β (PBL), murine IFN-α (PBL) and murine IL-6 (R&D). The mouse STING sequence was amplified by PCR using cDNA from BMDCs and subsequently cloned into a pcDNA3 vector (Invitrogen). All TRIM30α and STING deletion mutants/point mutants were constructed by PCR and subcloned into a pcDNA3 vector. The other plasmids were either generated or obtained as described previously [22,31].

### Cell culture, transfection and stimulation

D2SC cells were provided by Yong-Jun Liu (UT MD Anderson Cancer Center, Texas, USA) and maintained in Iscove’s modified Dulbecco’s medium containing 10% (vol/vol) heat-inactivated FCS and 1% (vol/vol) penicillin-streptomycin (Invitrogen-Gibco). L929, Vero, MEF, HEK293T and HEK293 cells were cultured in DMEM supplemented with 10% (vol/vol) FBS, penicillin (100 U/ml) and streptomycin (100 U/ml). Single-cell suspensions of CD11c^+^ splenocytes were isolated from spleens with CD11c MicroBeads (130-052-001; Miltenyi Biotec) following the manufacturer’s instructions. The procedure for generating BMDCs has been described previously [32]. X-tremeGENE was used for transient transfection of plasmid DNA into L929 and MEF. Transfection of 293T was performed with Turbofect. For stimulation, poly(dA:dT) (1 μg/ml), ISD (1 μg/ml), c-di-GMP (8 μg/ml), genomic DNA (2 μg/ml) or poly(I:C) (5 μg/ml) were delivered into cells using Lipofectamine 2000.

### Viruses and infection

HSV-1 and VACV were kindly provided by Xuetao Cao (Second Military Medical University, Shanghai, China) and Zhengfan Jiang (Peking University, Shanghai, China), respectively. Cells were infected with HSV-1 (MOI 10) or VACV (MOI 10) for 1.5 h and subsequently washed with PBS and cultured in fresh media. Cytokine production was analyzed 16 h or 24 h later. For the in vivo cytokine production study, age- and sex-matched groups of mice were intravenously infected with HSV-1 (1.2×10^7^ PFU per mouse). HSV-1 viral titer was determined by the plaque-forming assay on Vero cells.

### RNA-mediated interference

The TRIM30α siRNA T3 and negative control siRNA have been described previously [22]. D2SC cells were transfected with siRNA delivered by Lipofectamine 2000. At 24 h after transfection, the cells were used for further experiments.

### Real-time PCR

Total RNA was extracted from cells or tissues using TRIzol reagent (Invitrogen). The RNA was then used in a RT reaction using the Prime Script RT Master Mix kit (TaKaRa). All gene transcripts were analyzed by quantitative PCR with SYBR Green

Master Mix (ABI) using an ABI PRISM 7900HT Sequence Detection System (PE Applied Biosystems).

Primers for PCR are listed as follows:

IP-10:

sp 5’-GGGCCAGTGAGAATGAGGG-3’,

as 5’-GCTCGCAGGGATGATTTCAA-3’;

HSV-1 genomic DNA:

sp 5’-TGGGACACATGCCTTCTTGG-3’,

as 5’-ACCCTTAGTCAGACTCTGTTACTTACCC-3’;

IFN-α1

sp 5’-CCTGAACATCTTCACATCAAAGGA-3’,

as 5’-AGCTGCTGGTGGAGGTCATT-3’;

IFN-β:

sp 5’-CCTGGAGCAGCTGAATGGAA-3’,

as 5’-TTGAAGTCCGCCCTGTAGGT-3’;

TNF-α:

sp 5’-AAGCCTGTAGCCCACGTCGTA-3’,

as 5’-GGCACCACTAGTTGGTTGTCTTTG-3’;

ISG12

sp 5’-TTGCCAATGGAGGTGGAGTT-3’,

as 5’-AGGACCCCTGCTGATTGGA-3’;

ISG20

sp 5’-CGCTGCAGCATTGTGAACA-3’,

as 5’-CGGGTCGGATGTACTTGTCA-3’;

ISG56

sp 5’-CTCAGAGCAGGTCCAGTTCCTT-3’,

as 5’-GGCCAGGAGGTTGTGCAT-3’;

HPRT:

sp 5’-TGCTCGAGATGTCATGAAGGAG-3’,

as 5’-CAGAGGGCCACAATGTGATG-3’;

Acta2:

sp 5’-ATGACCCAGATTATGTTTGAGACC-3’;

as 5’-CCAGAGTCCAGCACAATACC-3’.

### In vitro ubiquitination assay

TRIM30α and STING proteins were expressed with a TNT Quick-coupled Transcription/Translation Systems kit (Promega). In vitro ubiquitination assay was performed with a ubiquitination kit (Enzo Life Science) following the manufacturer’s instructions.

### Immunoprecipitation and immunoblot analysis

These experiments were performed as described previously [22]. In brief, HEK293T cells were transfected with various combinations of plasmids. At 24 h after transfection, lysates of the cells were prepared in lysis buffer and incubated with the Protein A/G Plus-Agarose immunoprecipitation reagent together with the indicated Ab overnight at 4°C. Complexes were washed three times with lysis buffer and analyzed by immunoblot. For endogenous co-immunoprecipitation experiments, lysates of D2SC cells stimulated with poly(dA:dT) for 12 h were incubated with anti-TRIM30α or rabbit IgG Ab and analyzed by immunoblot. For ubiquitination, BMDCs were stimulated with 1 μg/ml ISD for 8 h delivered by Lipofectamine 2000 and then collected for immunoblot analysis with anti-STING.

### Confocal microscopy

L929 cells were transfected with expressing plasmids for cyan fluorescent protein-labeled TRIM30α and yellow fluorescent protein-labeled STING. After 24 h, cells were stimulated for 4 h with 1 μg/ml poly(dA:dT), 1 μg/ml ISD or left unstimulated. After stimulation, cells were fixed with 4% PFA in PBS and permeabilized with Triton X-100 and then blocked with 10% FBS in PBS, stained with anti-calnexin, followed by Alexa Fluor 647 donkey anti-rabbit IgG. Nuclei were stained with 4, 6-diamidino-2-phenylindole, and fluorescent images were captured with a Leica TCS SP2 laser confocal microscope.

### Luciferase reporter gene assay

MEF cells were transfected with an IFN-β luciferase reporter plasmid and a *Renilla* luciferase plasmid as an internal control plus the indicated expression plasmids. Empty control vector was added so that a total of 1 μg of DNA was transfected into each well of cells. Then, 24 h after transfection, cells were lysed, and reporter activity was analyzed with the Dual-Luciferase Reporter Assay system (Promega).

### Statistics

The data are presented as the mean ± SEM from at least three independent experiments. Student’s t-test was used to compare two independent groups. For all tests, values of *p* < 0.05 were considered statistically significant.

### Proteins accession numbers

The accession numbers in the UniProtKB/SwissProt database for the proteins in the manuscript are followed: TRIM30α, P15533; IFN-α, P01572; IFN-β, P01575; IL-6, P08505; IRF3, P70671; p65, Q04207; STING, Q3TBT3; TBK1, Q9WUN2; MDA5,Q9BYX4; VISA, Q7Z434.

## Supporting Information

S1 Fig TRIM30α protein is induced by cytoplasmic nucleic acid and TRIM30α knockdown promotes type I IFN and IL-6 production. (A) Immunoblot analysis of TRIM30α in lysates from BMDCs stimulated for 2–12 h with poly(dA:dT) (1 μg/ml). (B and C) Real-time PCR of IFN-α1, IFN-β and IL-6 mRNA in D2SC cells treated with siRNA SC or T3 and then stimulated for 8 h with poly(dA:dT) (1 μg/ml), ISD (1 μg/ml) or poly(I:C) (5 μg/ml) (B) or infected for 4 h with HSV-1 (MOI 10). (D) Immunoblot analysis of TRIM30α in lysates of BMDCs stimulated for 16 h with poly(I:C) (5 μg/ml), poly(dA:dT) (1 μg/ml), ISD (1 μg/ml), c-di-GMP (8 μg/ml) and genomic DNA (2 μg/ml) or infected for 16 h with HSV-1 (MOI 10) or VACV (MOI 10). (E) Immunoblot analysis of TRIM30α in lysates from BMDCs stimulated for 6 h with poly(I:C) (5 μg/ml), ISD (1 μg/ml) and HSV-1 (MOI 10), pretreated for 1 h with the indicated signaling inhibitors, 10 μM TPCA-1 and 100 μM PDTC. The data are representative of three independent experiments and are presented as mean ± SEM. *p < 0.05, **p < 0.01 and ***p < 0.001.
(TIF)

S2 Fig Generation of *Trim30α*-deficient mice. (A) The design of the *Trim30α* knockout mouse is shown. Exon 2 was knocked out by homologous recombination. (B) PCR genotyping of *Trim30α* knockout mice. (C) Photos of 6-week-old male WT and *Trim30α* ^-/-^ littermates. (D) Genotypes of the offsprings from the breeding of *Trim30α* heterozygous mice.
(TIF)

S3 Fig TRIM30α knockdown or deficiency enhances IFN-β and IL-6 production against RNA virus. (A and B) ELISA of IFN-β and IL-6 in D2SC cells treated with siRNA SC or T3 for 24 h, or in wild type (WT) and *Trim30α* ^-/-^ (KO) BMDCs mock treated or infected with VSV (MOI 1) for 16 h. The data are representative of three independent experiments and are presented as mean ± SEM. *p < 0.05, **p < 0.01 and ***p < 0.001.
(TIF)

S4 Fig TRIM30α deficiency promotes production type I IFN and ISGs production in peritoneal macrophages (PM). (A and B) Real-time PCR of IFN-α1, IFN-β (A), ISG12, ISG20, ISG56 and IP-10 mRNA in peritoneal macrophage from WT and *Trim30α* ^-/-^ mice treated with ISD (1 μg/ml) or HSV-1 (MOI 10) for 8 h. The data are representative of three independent experiments and are presented as mean ± SEM. *p < 0.05, **p < 0.01 and ***p < 0.001.
(TIF)

S5 Fig TRIM30α interacts with STING in vitro. HA-tagged STING and Myc-tagged vector or TRIM30α were quickly translated in vitro, and STING and TRIM30α proteins were mixed together, followed by immunoprecipitation with anti-HA, and analyzed via immunoblot with anti-Myc.
(TIF)
