# Supplementary figures and images for: TRIM30α Is a Negative-Feedback Regulator of the Intracellular DNA and DNA Virus-Triggered Response by Targeting STING
Source: PLoS Pathog. 2015 Jun 26;11(6):e1005012. doi: 10.1371/journal.ppat.1005012 (PMC4482643; doi:10.1371/journal.ppat.1005012)

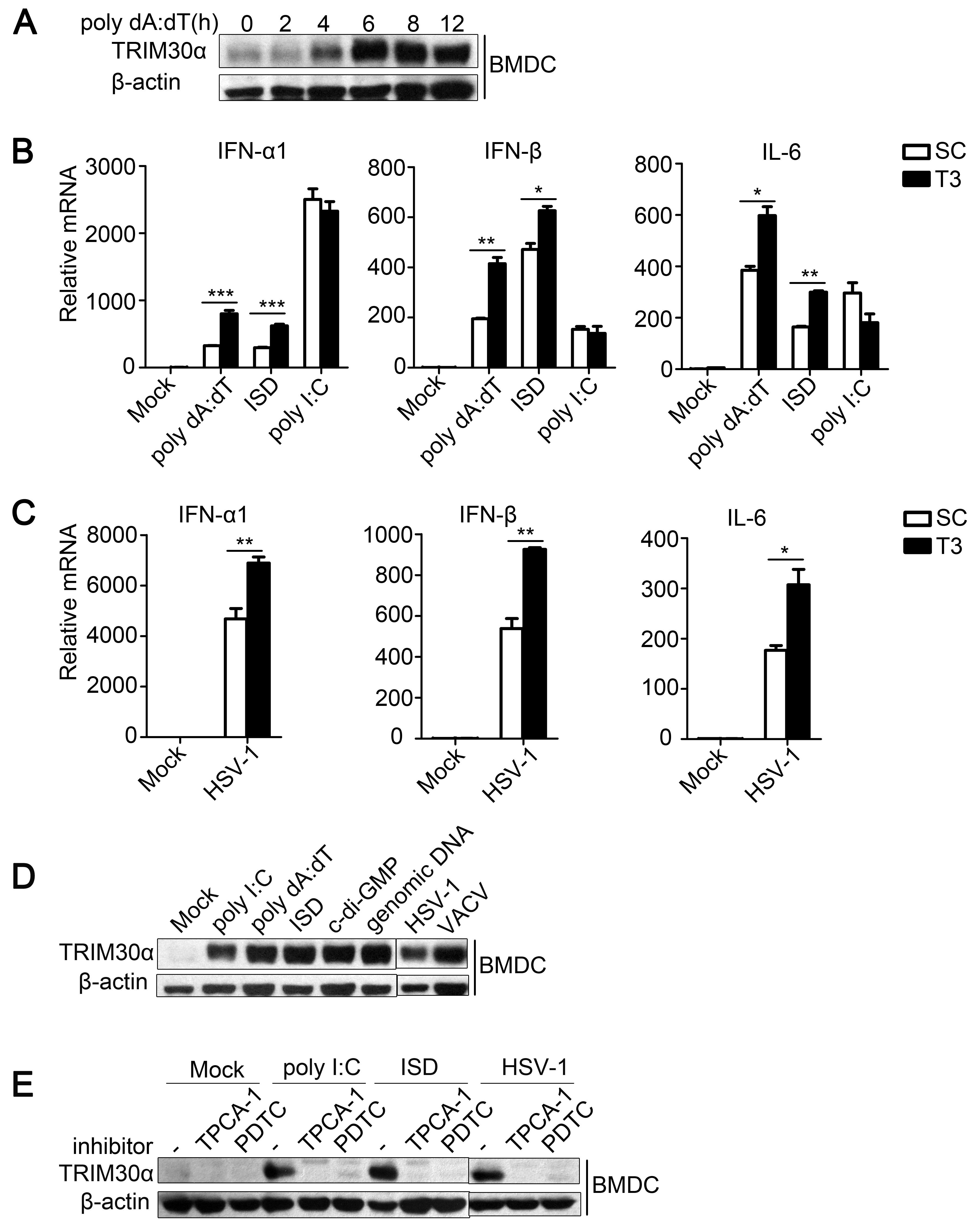

Supplement: S1 Fig — (A) Immunoblot analysis of TRIM30α in lysates from BMDCs stimulated for 2–12 h with poly(dA:dT) (1 μg/ml). (B and C) Real-time PCR of IFN-α1, IFN-β and IL-6 mRNA in D2SC cells treated with siRNA SC or T3 and then stimulated for 8 h with poly(dA:dT) (1 μg/ml), ISD (1 μg/ml) or poly(I:C) (5 μg/ml) (B) or infected for 4 h with HSV-1 (MOI 10). (D) Immunoblot analysis of TRIM30α in lysates of BMDCs stimulated for 16 h with poly(I:C) (5 μg/ml), poly(dA:dT) (1 μg/ml), ISD (1 μg/ml), c-di-GMP (8 μg/ml) and genomic DNA (2 μg/ml) or infected for 16 h with HSV-1 (MOI 10) or VACV (MOI 10). (E) Immunoblot analysis of TRIM30α in lysates from BMDCs stimulated for 6 h with poly(I:C) (5 μg/ml), ISD (1 μg/ml) and HSV-1 (MOI 10), pretreated for 1 h with the indicated signaling inhibitors, 10 μM TPCA-1 and 100 μM PDTC. The data are representative of three independent experiments and are presented as mean ± SEM. *p < 0.05, **p < 0.01 and ***p < 0.001. (TIF) [file ppat.1005012.s001.tif]

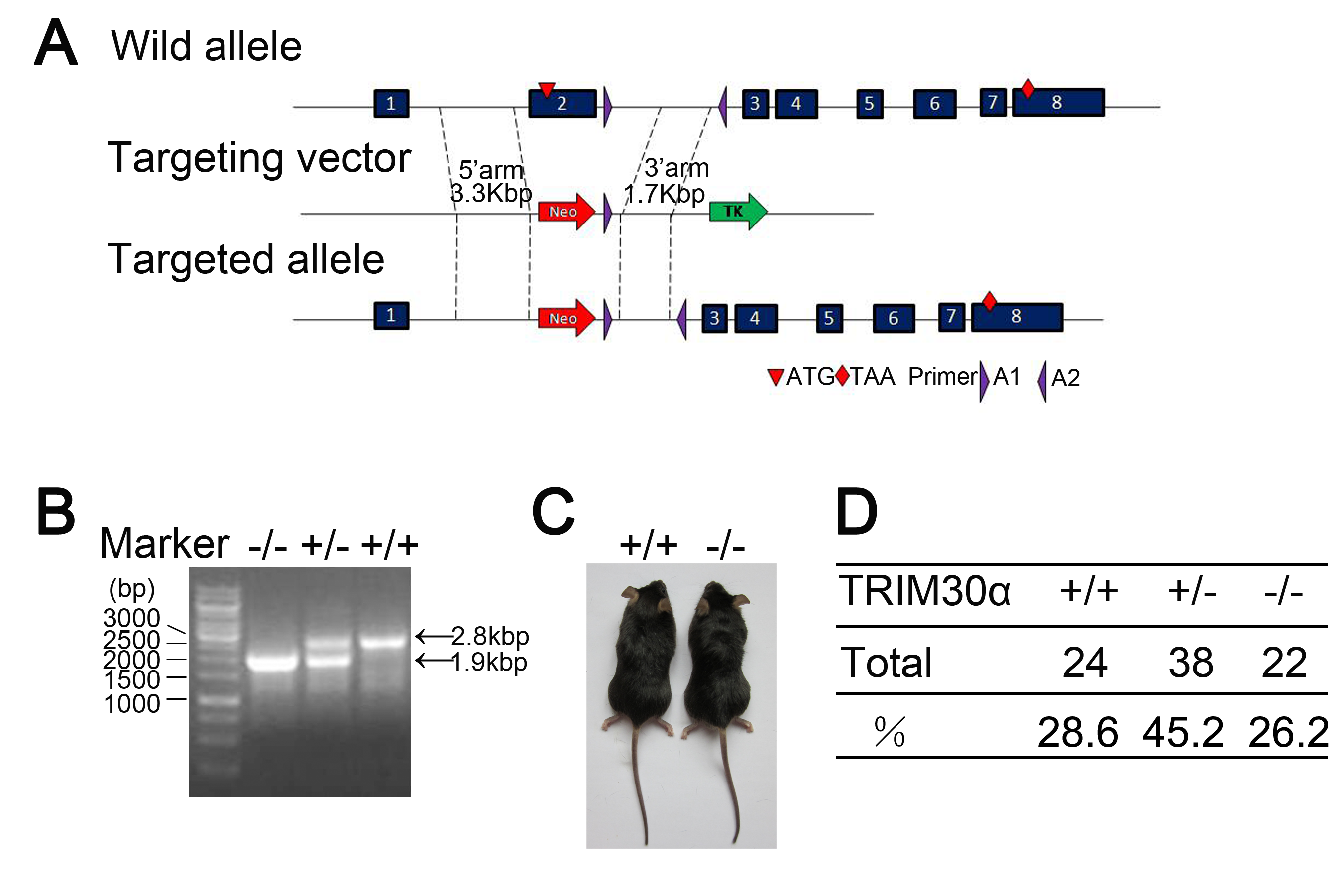

Supplement: S2 Fig — (A) The design of the Trim30α knockout mouse is shown. Exon 2 was knocked out by homologous recombination. (B) PCR genotyping of Trim30α knockout mice. (C) Photos of 6-week-old male WT and Trim30α -/- littermates. (D) Genotypes of the offsprings from the breeding of Trim30α heterozygous mice. (TIF) [file ppat.1005012.s002.tif]

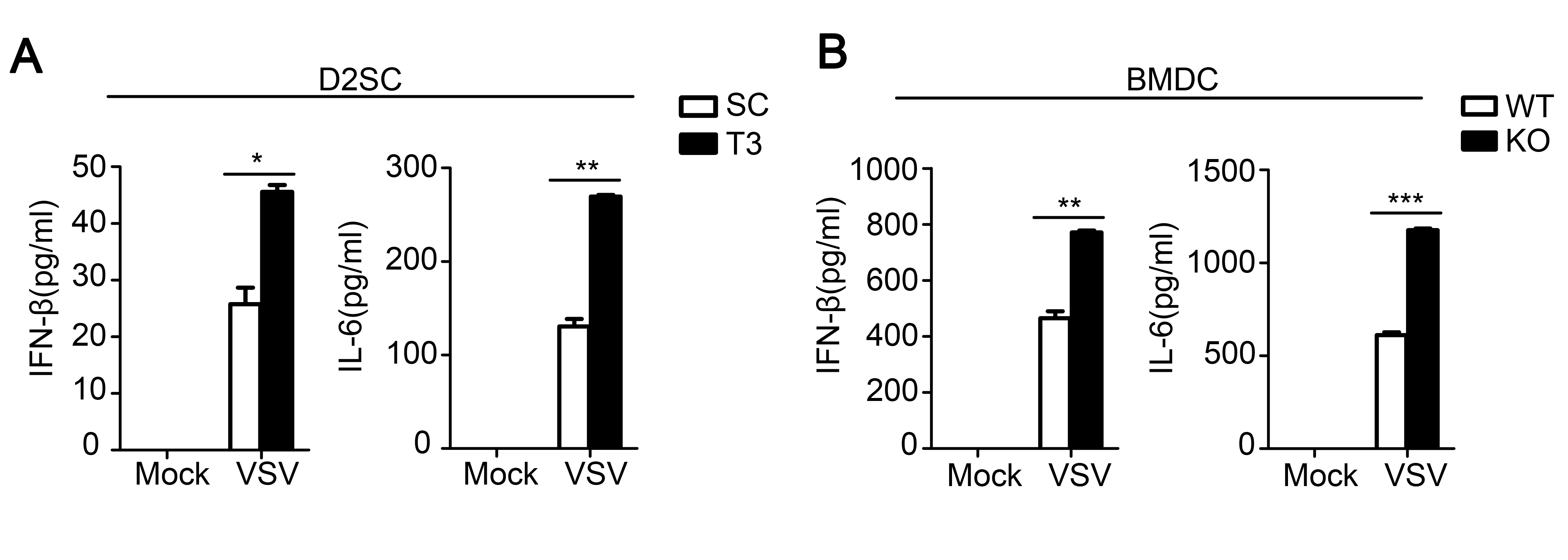

Supplement: S3 Fig — (A and B) ELISA of IFN-β and IL-6 in D2SC cells treated with siRNA SC or T3 for 24 h, or in wild type (WT) and Trim30α -/- (KO) BMDCs mock treated or infected with VSV (MOI 1) for 16 h. The data are representative of three independent experiments and are presented as mean ± SEM. *p < 0.05, **p < 0.01 and ***p < 0.001. (TIF) [file ppat.1005012.s003.tif]

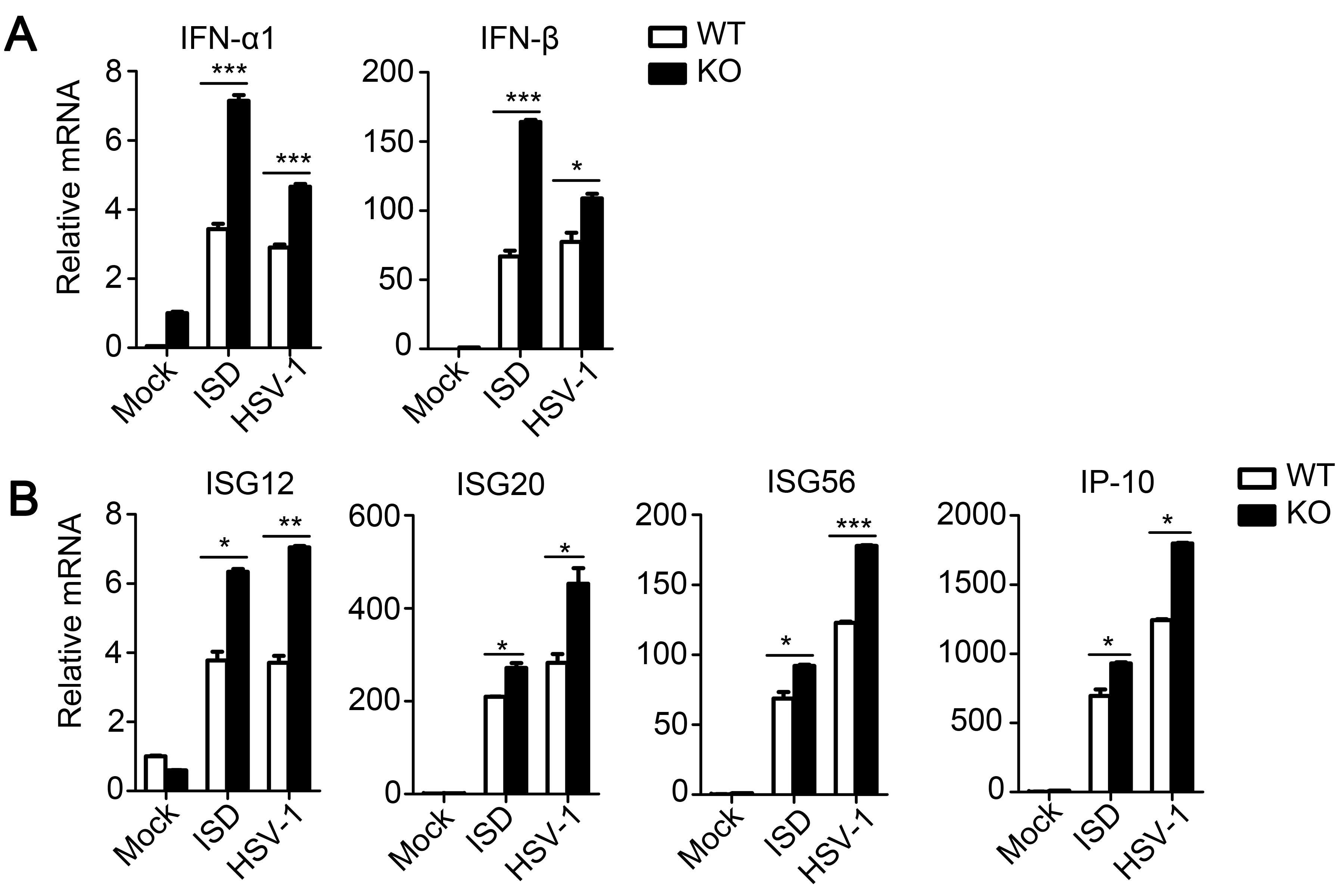

Supplement: S4 Fig — (A and B) Real-time PCR of IFN-α1, IFN-β (A), ISG12, ISG20, ISG56 and IP-10 mRNA in peritoneal macrophage from WT and Trim30α -/- mice treated with ISD (1 μg/ml) or HSV-1 (MOI 10) for 8 h. The data are representative of three independent experiments and are presented as mean ± SEM. *p < 0.05, **p < 0.01 and ***p < 0.001. (TIF) [file ppat.1005012.s004.tif]

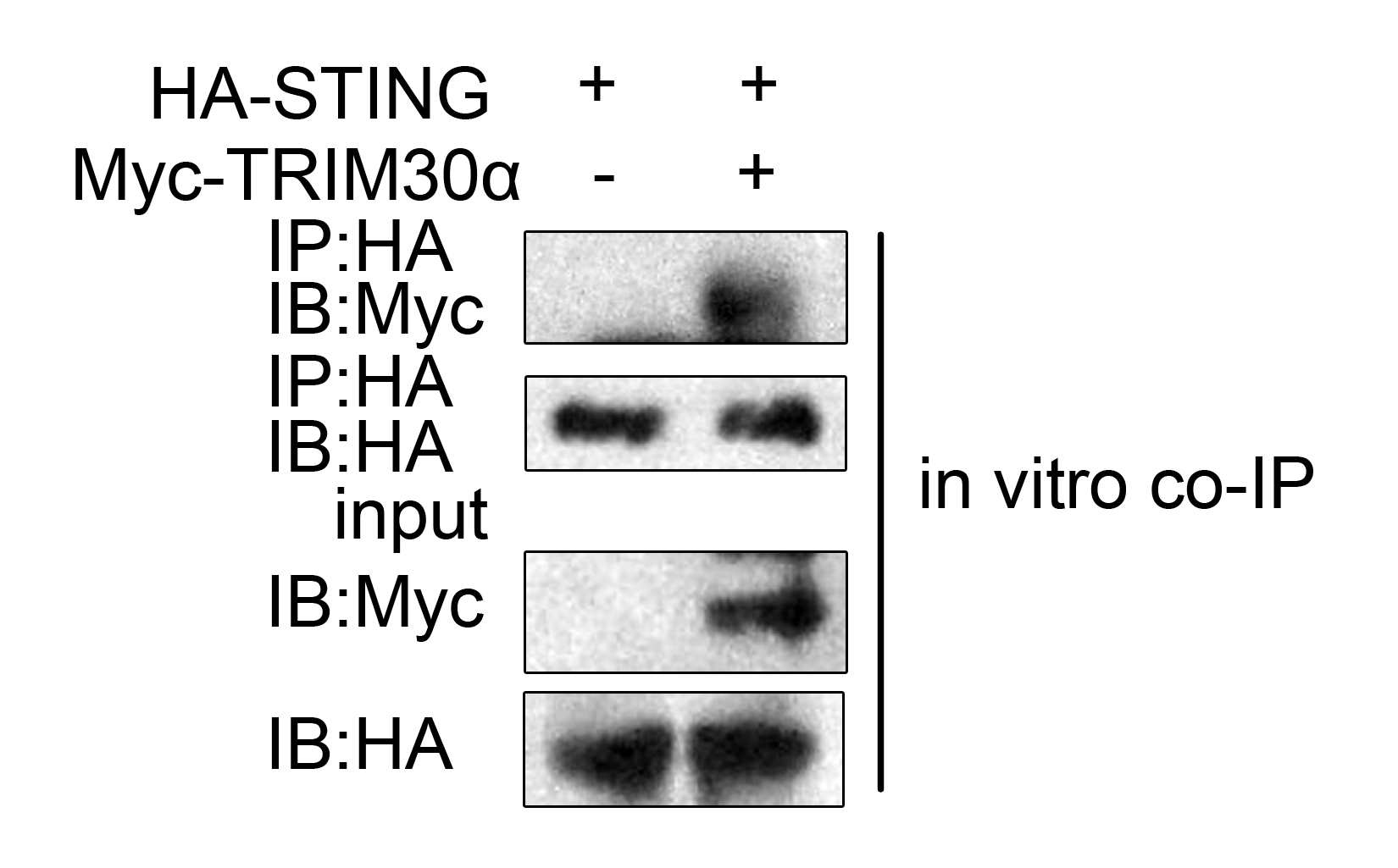

Supplement: S5 Fig — HA-tagged STING and Myc-tagged vector or TRIM30α were quickly translated in vitro, and STING and TRIM30α proteins were mixed together, followed by immunoprecipitation with anti-HA, and analyzed via immunoblot with anti-Myc. (TIF) [file ppat.1005012.s005.tif]
